# Metabolic engineering of terpene biosynthesis in plants using a trichome‐specific transcription factor *MsYABBY5* from spearmint (*Mentha spicata*)

**DOI:** 10.1111/pbi.12525

**Published:** 2016-02-04

**Authors:** Qian Wang, Vaishnavi Amarr Reddy, Deepa Panicker, Hui‐Zhu Mao, Nadimuthu Kumar, Chakravarthy Rajan, Prasanna Nori Venkatesh, Nam‐Hai Chua, Rajani Sarojam

**Affiliations:** ^1^ Temasek Life Sciences Laboratory1 Research LinkNational University of SingaporeSingapore CitySingapore; ^2^ Department of Biological SciencesNational University of SingaporeSingapore CitySingapore; ^3^ Laboratory of Plant Molecular BiologyThe Rockefeller UniversityNew YorkNYUSA; ^4^ Present address: Singapore Centre on Environmental Life Sciences EngineeringNanyang Technological UniversitySingapore637551Singapore

**Keywords:** spearmint, secondary metabolism, terpene, sweet basil, transcription factor, YABBY

## Abstract

In many aromatic plants including spearmint (*Mentha spicata*), the sites of secondary metabolite production are tiny specialized structures called peltate glandular trichomes (PGT). Having high commercial values, these secondary metabolites are exploited largely as flavours, fragrances and pharmaceuticals. But, knowledge about transcription factors (TFs) that regulate secondary metabolism in PGT remains elusive. Understanding the role of TFs in secondary metabolism pathway will aid in metabolic engineering for increased yield of secondary metabolites and also the development of new production techniques for valuable metabolites. Here, we isolated and functionally characterized a novel *MsYABBY5* gene that is preferentially expressed in PGT of spearmint. We generated transgenic plants in which *MsYABBY5* was either overexpressed or silenced using RNA interference (RNAi). Analysis of the transgenic lines showed that the reduced expression of *MsYABBY5* led to increased levels of terpenes and that overexpression decreased terpene levels. Additionally, ectopic expression of *MsYABBY5* in *Ocimum basilicum* and *Nicotiana sylvestris* decreased secondary metabolite production in them, suggesting that the encoded transcription factor is probably a repressor of secondary metabolism.

## Introduction

The genus *Mentha*, a member of *Lamiaceae* family, includes species that are widely used as medicinal and aromatic herbs. The essential oils produced by these plants find wide usage in food, flavour, cosmetic and pharmaceutical industries (Champagne and Boutry, 2013; Sinha *et al*., 2013). Plants produce these volatile essential oils as secondary metabolites which have important roles in plant defence, plant‐to‐plant communication and pollination (Gershenzon *et al*., 2000). In mint, these essential oils are produced in specialized nonphotosynthetic glandular trichomes known as peltate glandular trichomes (PGT) which are found on the aerial surface of the plants. The PGT are dedicated to the production and storage of large amounts of volatile secretions (Champagne and Boutry, 2013; Croteau *et al*., 2000; Lange and Turner, 2013). In the case of spearmint (*Mentha spicata*), the essential oil is dominated mainly by two monoterpenes, limonene and carvone. Monoterpenes are the C10 type of terpenoids and are generally colourless, lipophilic and volatile. They are responsible for the characteristic aromas and flavours of essential oils, floral scents and resin of aromatic plants (Loza‐Tavera, 1999). Given their economic importance, strategies to metabolically engineer monoterpenes to increase yield is of considerable interest.

Varietal improvement in cultivated spearmint or peppermint varieties has been challenging because these varieties are sterile hybrids making classical breeding approach unfeasible. Hence, metabolic engineering provides an alternative method to improve essential oil yield and composition. Plants synthesize terpenes either by the mevalonate (MVA) pathway in the cytosol or by the 2‐C‐methyl‐d‐erythritol 4‐phosphate (MEP) pathway in plastids. Both pathways provide the precursors for terpene biosynthesis (Vranová *et al*., 2013) and have been well investigated. The MEP pathway in plastids is mainly responsible for producing monoterpenes and diterpenes, whereas the MVA pathway generates sesquiterpenes and triterpenes (Dubey *et al*., 2003). Studies have shown that under certain conditions an exchange of precursor metabolites can occur between the cytosolic MVA and plastid MEP pathways. Analysis of terpenoid production in many different plants has shown that under specific ecological conditions synthesis of monoterpenes, diterpenes, sesquiterpenes and polyterpenes can occur from precursors produced by both pathways. Metabolic intermediates like isopentenyl diphosphate, geranyl diphosphate or geranylgeranyl diphosphate can be transported across plastidial membranes (Hemmerlin *et al*., 2012; Vranová *et al*., 2013).

Apart from the precursor pathways, the downstream monoterpene biosynthetic pathways in both spearmint and peppermint are also well characterized (Lange *et al*., 2011). One of the strategies to increase yield in peppermint was to manipulate genes that code for enzymes involved in the monoterpene pathway, for example genes for limonene synthase and limonene hydroxylase (Diemer *et al*., 2001; Mahmoud *et al*., 2004), but overexpression of these genes did not enhance oil yields significantly. In their pioneering work, Mahmoud and Croteau (2001) and Lange *et al*. (2011) evaluated the efficacy of overexpressing genes encoding enzymes involved in precursor pathways on oil yields in peppermint (*Mentha piperita*). Most encouraging results were obtained in plants where two genes were manipulated simultaneously, the gene encoding 1‐deoxy‐d‐xylulose‐5‐phosphate reductoisomerase was overexpressed and the gene encoding menthofuran synthase was down‐regulated. Oil yields in these transgenic plants increased up to 61% over wild‐type controls while reducing the undesirable side‐products (+)‐menthofuran and its intermediate (+)‐pulegone (Lange *et al*., 2011). Recently, increase in monoterpene formation was achieved by introducing a noncanonical substrate neryl diphosphate (NPP), but the additional NPP had to be eliminated to avoid adverse impact on plant growth (Gutensohn *et al*., 2014).

It is increasingly evident that transcription factors (TFs) which are regulators of structural genes can activate or repress multiple genes in a metabolic pathway (Grotewold, 2008; Iwase *et al*., 2009). Manipulation of such TFs can be more effective for engineering pathways rather than changing the expression of genes for individual enzymes involved, because plant metabolic pathways are complex comprising of multiple genes encoding various enzymes (Broun and Somerville, 2001). The effectiveness of using TFs to modulate metabolic pathways has been validated in a few studies (Butelli *et al*., 2008; Luo *et al*., 2008; Schwinn *et al*., 2006). Although the enzymatic pathway leading to the synthesis of spearmint monoterpenes is well defined (Croteau *et al*., 1991; Lange *et al*., 2011; Muñoz‐Bertomeu *et al*., 2008), the developmental regulation of this secondary metabolite pathway still remains elusive. Few TFs have been reported from other plants that are involved in regulating terpene biosynthesis. They are *Artemisia annua*,* AaWRKY1, AaERF1, AaERF2, AaORA1* and *AabZIP1* (Lu *et al*., 2013; Ma *et al*., 2009; Yu *et al*., 2012; Zhang *et al*., 2015), cotton *GaWRKY1* (Xu *et al*., 2004), *TaWRKY1* from *Taxus chinensis* (Wang *et al*., 2001), rubber *EREBP1* and *HbWRKY1* (Chen *et al*., 2012; Zhou *et al*., 2012) and *OsTGAP1* in rice (Miyamoto *et al*., 2014).

To investigate the genes involved in PGT formation and secondary metabolism in spearmint, we had performed comparative RNA‐Seq analysis of different tissues of spearmint, namely PGT, leaf devoid of PGT (leaf‐PGT) and leaf in a previous study (Jin *et al*., 2014). This led to the identification of many TF transcripts that were significantly more abundant in PGT when compared to leaf‐PGT and a *YABBY* transcript was among the top candidates. We cloned the full‐length cDNA of this transcript and sequence analysis showed that it is similar to YABBY5 subfamily of proteins*. YABBY* genes constitute a group of plant‐specific TFs that are known to play important roles in various aspects of vegetative and floral development in plants (Bonaccorso *et al*., 2012; Bowman, 2000). In this study, we report the engineering of spearmint plants for higher yields by suppressing this glandular trichome‐enriched TF *MsYABBY5*. The resulting *MsYABBY5* RNAi lines showed an increase in monoterpene production which ranged from 20% to 77%. This is the first report of a transcription factor regulating monoterpene production in mint plants and assigns a new role for *YABBY* genes in plant secondary metabolism. Ectopic expression of *MsYABBY5* in sweet basil (*Ocimum basilicum*), an aromatic herb similar to mint, and in *Nicotiana sylvestris* resulted in decreased secondary metabolite production in them. Essential oil of sweet basil has compounds derived from both terpene and phenylpropanoid pathways, whereas *N. sylvestris* produces mainly diterpenes. As MsYABBY5 could affect metabolites derived from different metabolic pathways, it suggests that it regulates an upstream step in plant secondary metabolism. We further found that MsYABBY5 probably regulates terpene synthesis through a regulatory network that involves *MsWRKY75*.

## Results

### *MsYABBY5* shows high expression in spearmint PGT

**Figure 1:**
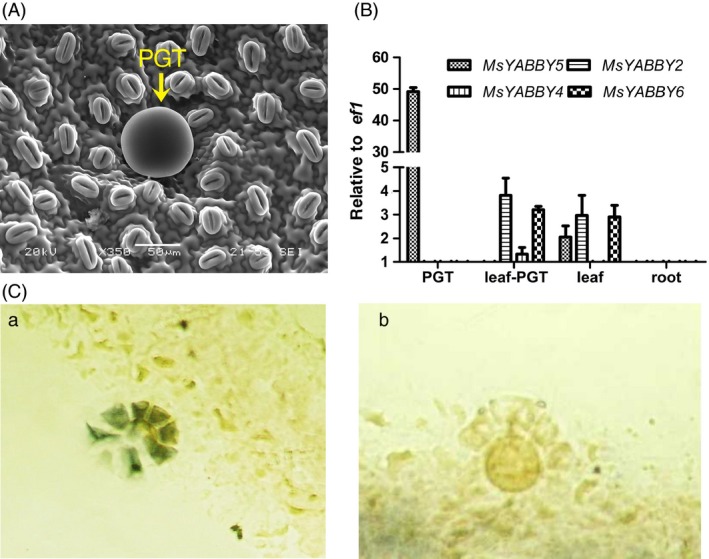
Validation of *MsYABBY* genes expression pattern in spearmint. (A) Spearmint leaf showing peltate glandular trichome (PGT) on upper leaf surface as visualized under scanning electron microscope. (B) qRT‐PCR analysis of *MsYABBY* genes in different tissues. PGT, peltate glandular trichome; leaf‐PGT, leaves where PGT were brushed away. The housekeeping gene *elongation factor 1 (ef1)* was used as control. (C) *In situ* hybridization: antisense (a) and sense (b) probe detection of *MsYABBY5*.

**Figure 2:**
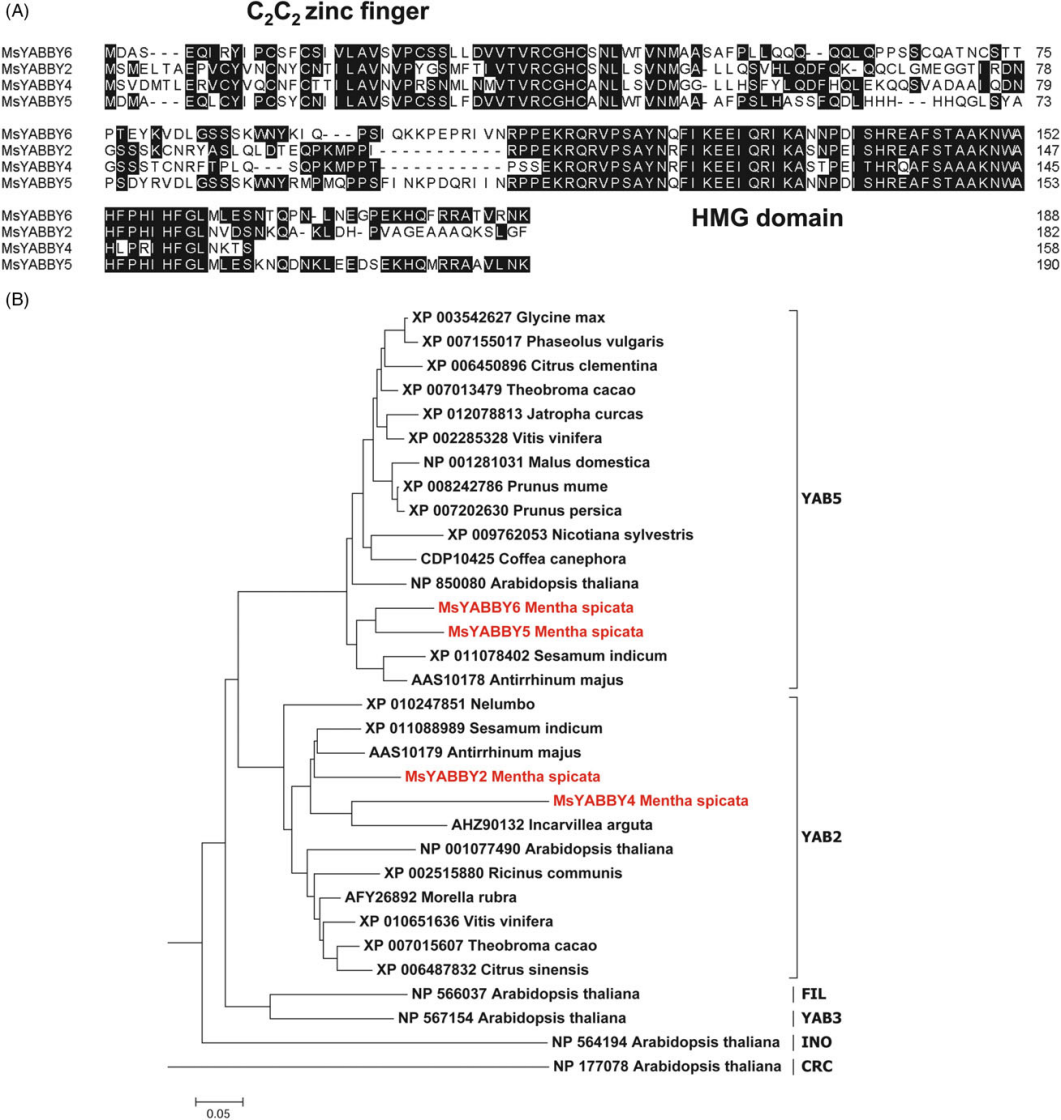
Amino acid sequence alignment (A) and phylogenetic tree analysis (B) of MsYABBYs.

Mint leaves have PGT on both surfaces (Figure 1A). From the RNA‐Seq data of leaves, we identified four *YABBY*‐like transcripts that showed high expression levels. Of these, only *MsYABBY5* was preferentially expressed in PGT, whereas the others were more enriched in leaf tissues. The differential expression pattern of these transcripts as observed by RNA Seq was further validated by quantitative RT‐PCR (qRT‐PCR) (Figure 1B). Full‐length open reading frames (ORFs) of all these four *YABBYs* including *MsYABBY5* were amplified from leaf cDNA using RACE. All the four cloned ORFs contained a conserved C_2_C_2_ zinc finger domain located at N‐terminus and a helix‐loop‐helix motif (YABBY domain) at the C terminus which is similar to the HMG box motif. These two domains are highly conserved among all YABBY proteins (Figure 2A). As we were interested in TFs involved in regulating secondary metabolism in mint, we focussed on *MsYABBY5*. *In situ* hybridization also confirmed the PGT‐specific expression of *MsYABBY5*, as no signal was observed in the leaf tissue (Figure 1C). The ORF of *MsYABBY5* encoded a polypeptide of 190 amino acids. BLAST analysis showed that *MsYABBY5* has highest sequence similarity to *Antirrhinum PROLONGATA YABBY*‐like transcription factor. We generated a phylogenetic tree based on amino acids sequences of YABBY proteins from different plants. The results revealed that MsYABBY5 and MsYABBY6 belonged to the YABBY5 subfamily, whereas the other two, MsYABBY2 and MsYABBY4*,* are members of the YABBY2 subfamily (Figure 2B).

### Subcellular localization of MsYABBY5 protein

**Figure 3:**
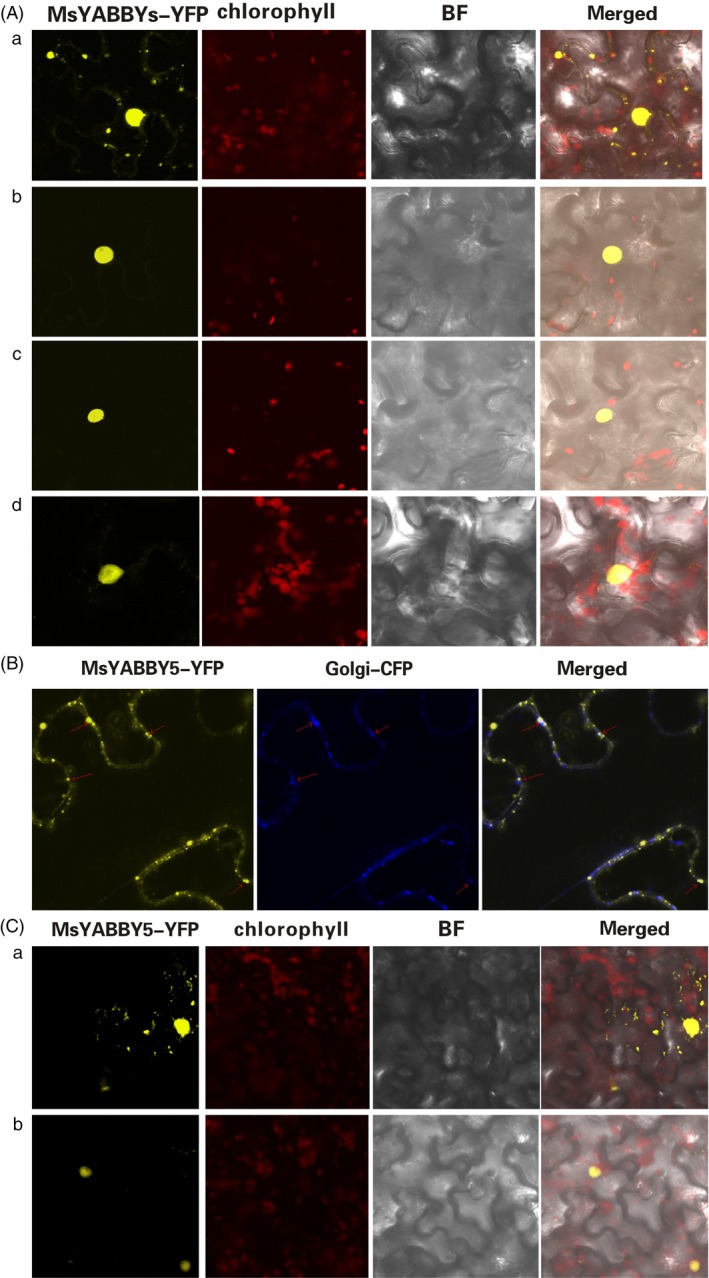
Subcellular localization of MsYABBYs in *Nicotiana benthamiana**.*** (A) MsYABBY5 showed both nuclear and cytoplasmic expression, while other MsYABBYs were found in nucleus only. (a) MsYABBY5 was localized to both nucleus and cytoplasm. (b) MsYABBY6. (c) MsYABBY2. (d) MsYABBY4. (B) MsYABBY5 protein colocalization with Golgi marker. (C) BFA treatment leads to nuclear localization of MsYABBY5 protein in *N. benthamiana*. (a) Mock group treated with DMSO. (b) Test group treated with 50 μg/mL BFA for 3 h.

To examine the subcellular localization patterns of YABBY proteins, cDNAs of all the four *MsYABBYs* were fused in‐frame to cDNA encoding the yellow‐fluorescent protein (YFP) and the fusion genes were transiently expressed in tobacco by agroinfiltration. All the MsYABBYs except MsYABBY5 showed nuclear localization. Interestingly, MsYABBY5 showed both nuclear and cytoplasmic localization (Figure 3A). Online software prediction programs indicated that MsYABBY5 contained a potential transmembrane domain (http://dgpred.cbr.su.se/index.php?p=fullscan) at the amino terminal and participated in the secretory pathway (http://www.cbs.dtu.dk/services/TargetP-1.1/output.php). To investigate this, Golgi markers were used for colocalization experiment which showed that MsYABBY5 localized to Golgi (Figure 3B). To further assess this localization pattern, tobacco leaves were treated with Brefeldin A (BFA). BFA treatment in tobacco results in the complete disappearance of Golgi apparatus and disrupts the secretory system (Robinson and Ritzenthaler, 2006). After treatment with 50 μg/mL BFA for 3 h, MsYABBY5 was found to exhibit nuclear localization only, while both nuclear and cytosolic distribution was still observed in the control plants (treated with 1 : 1000 dilution of DMSO in ddH_2_O) (Figure 3C).

To directly observe the distribution pattern of MsYABBY5 protein, we performed immunogold labelling of native MsYABBY5 in PGT of spearmint. A 14‐amino acid peptide that is presumably located on the surface of the predicted three‐dimensional structure of MsYABBY5 was used as an antigen to raise specific antibody. Western blot analysis showed that the antibody exclusively bound to MsYABBY5 but not to MsYABBY2, MsYABBY4 or MsYABBY6 (Figure S1A,B). MsYABBY5 proteins conjugated with gold particles were observed to accumulate inside the nucleus and also present in the cytoplasm. As the cell organelles were not clearly distinguishable in the TEM sections, the localization to cell organelles could not be verified by immunostaining (Figure S1C).

### Analysis of the promoter region of *MsYABBY5*

**Figure 4:**
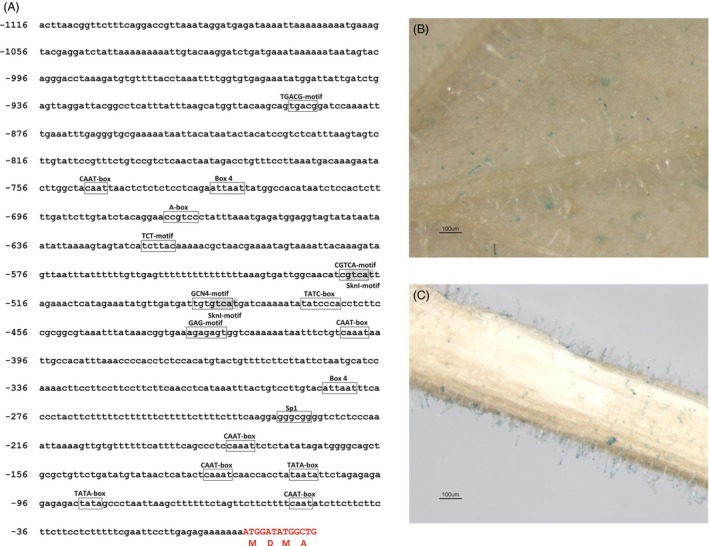
Ms*YABBY5* promoter analysis and expression pattern. (A) *cis‐*acting regulatory elements in the 5′UTR (−1116 bp) region of *MsYABBY5*. (B and C) Trichome‐specific GUS expression pattern observed in *Nicotiana benthamiana* leaves and stems *of* plants transformed with p*M* *sYABBY5::GUS*.

**Figure 5:**
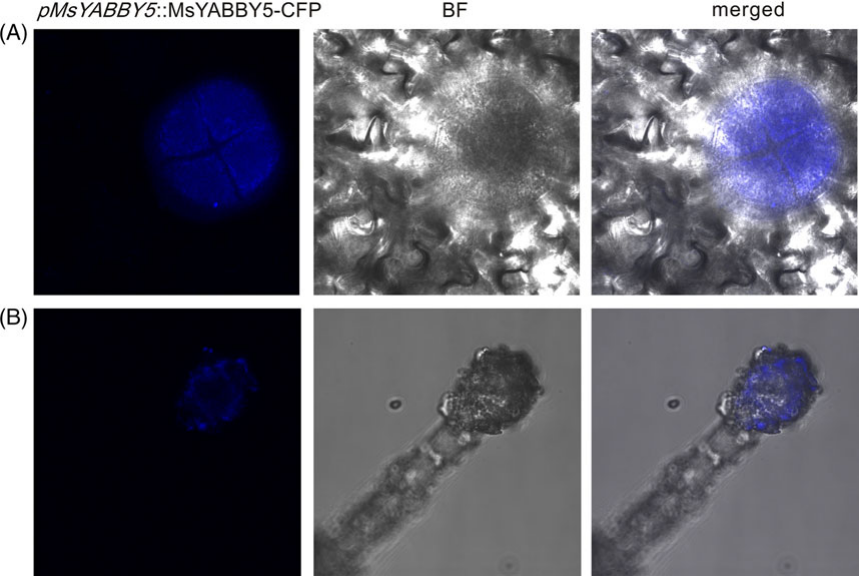
Localization of MsYABBY5 under its native promoter in sweet basil (A) and tobacco (B). p*M* *sYABBY5*:: MsYABBY5‐CFP showed exclusive expression in PGT of plants.

A 1116‐bp genomic DNA fragment upstream of the putative start codon of *MsYABBY5* was cloned by genome walking. Bioinformatics analysis of this region revealed the presence of many *cis*‐acting regulatory elements apart from the common TATA and CAAT box (http://bioinformatics.psb.ugent.be/webtools/plantcare/html/) (Figure 4A). Four light‐responsive motifs, including two Box4, one TCT and one Sp1 motifs, were found in the promoter sequence. Regulation of terpene biosynthesis by light is known in many plants (Cordoba *et al*., 2009). With respect to hormones, two *cis*‐acting elements, CGTCA‐motif and TGACG‐motif, involved in the MeJA‐responsiveness were found and one for gibberellin *cis* elements, TATC box, was found within the sequence (Zhou *et al*., 2012; Zhu *et al*., 2014). Further, tissue‐specific expression pattern of the cloned promoter was analysed. The 1116‐bp promoter fragment was fused with a β‐glucuronidase (GUS) reporter gene and transformed into *Nicotiana benthamiana* plants. The transgenic plants showed trichome‐specific expression pattern in leaves and stems of tobacco plants (Figure 4B,C). No staining was observed in flowers or roots. Hence, this promoter is potentially a glandular trichome‐specific promoter. Additionally, this promoter was used to drive *MsYABBY5* cDNA fused to a cyan‐fluorescent protein reporter gene in basil and tobacco. The fluorescence was observed exclusively in PGT of basil plants and head cells of the glandular trichomes of tobacco, but subcellular localization was difficult to decipher (Figure 5).

### Silencing of *MsYABBY5* Increases monoterpene production in spearmint

**Figure 6:**
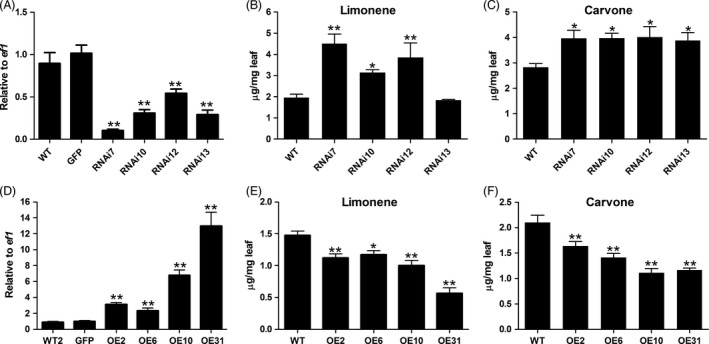
Transcript level of *MsYABBY5* and monoterpene production in *MsYABBY5* RNAi and overexpression plants. (A) *MsYABBY5* transcripts level in RNAi plants. (B) Limonene production in RNAi plants. (C) Carvone production in RNAi plants. (D) *MsYABBY5* transcripts level in overexpression plants. (E) Limonene production in overexpression plants. (F) Carvone production in overexpression plants. Gene expression is presented as relative to *ef1*. Leaves from the second node (2–3 cm) were harvested and used for analysis. Results of terpene production are presented as mean ± SD. *P < 0.05; **P < 0.01.

To examine the function of *MsYABBY5* in spearmint PGT, an RNAi construct targeting a specific region of *MsYABBY5* was generated and transformed into wild‐type spearmint using *Agrobacterium tumefaciens*‐mediated T‐DNA transfer. Many transgenic lines were generated, of which four independent transgenic lines analysed by Southern blotting for transgene integration were selected for further characterization (Figure S2). All these RNAi plants showed a reduction in *MsYABBY5* transcripts (Figure 6A). No significant changes were observed in the expression of other leaf‐specific *YABBY* genes (*MsYABBY2*,* MsYABBY4* and *MsYABBY6*), suggesting that the RNAi construct was specific to *MsYABBY5* and did not target other *YABBY* transcripts (Figure S3). The RNAi transgenic plants appeared phenotypically similar to WT plants. Scanning electron microscopy analysis revealed no phenotypical changes in either the number or the structure of PGT.

Gas chromatography–mass spectrometry (GC–MS) analysis was performed on these transgenic plants to evaluate the quality and quantity of the volatiles produced. Young WT spearmint leaves contain an abundance of both limonene and carvone monoterpenes. Limonene is the first committed step towards carvone production. Limonene is converted to carvone by a two‐step reaction. In our greenhouse conditions, we observed that in WT spearmint, the productions of limonene and carvone were about 1.47 ± 0.11 and 2.10 ± 0.25 μg/mg fresh leaf, respectively. Upon GC–MS analysis, all the four transgenic lines showed a significant increase (20%–77%) in total monoterpene production (limonene and carvone) (Figure 6B,C). The RNAi lines were tested for the expression levels of enzymes involved in carvone production (limonene synthase, limonene 6‐hydroxylase and carveol dehydrogenase) by qRT‐PCR; however, no major changes were observed. Transcripts for all enzymes of the MEP precursor pathways including geranyl diphosphate synthase small and big subunits were also investigated, but no significant changes in their expression levels were found. These results suggest that increase in monoterpene production is probably not due to the transcriptional activation of biosynthetic genes. *MsYABBY5* might be acting upstream to regulate flux into the terpene pathway.

### Overexpression of *MsYABBY5* results in decrease in monoterpene production

To gain further insight into the role of *MsYABBY5* in secondary metabolism, we overexpressed this gene in spearmint under the control of a CaMV *35S* promoter. Four independent lines confirmed by southern hybridization were selected for further characterization. The results of qRT‐PCR showed high *MsYABBY5* expression levels in all the transgenic plants (Figure 6D). GC–MS analysis of young leaves showed a reduction in total monoterpene production, which ranged from 23% to 52% (Figure 6E,F). The observed phenotype in RNAi and overexpression transgenic lines suggests that *MsYABBY5* might be a repressor of secondary metabolism in spearmint.

### Possible downstream target of MsYABBY5 to regulate secondary metabolism

**Figure 7:**
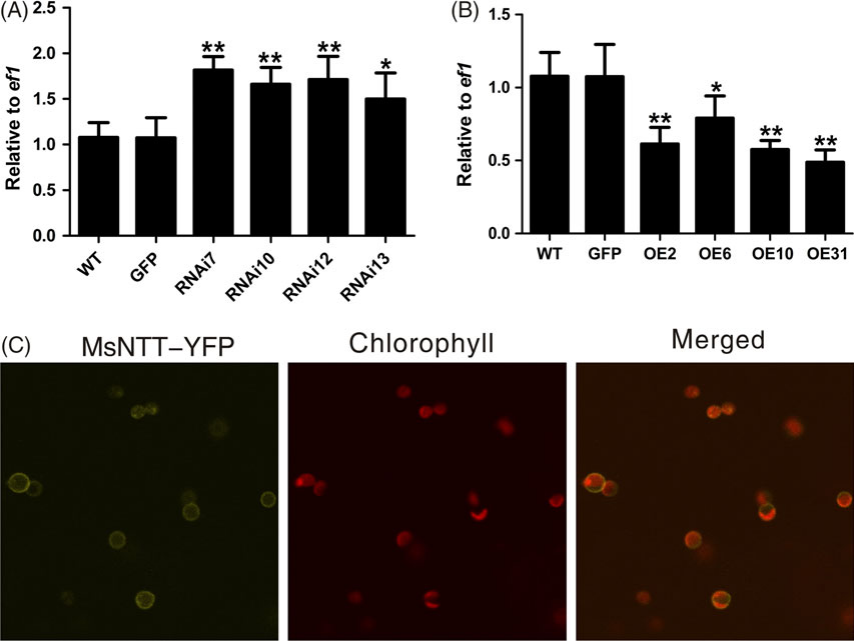
*MsNTT* expression and localization. Transcript levels of *MsNTT* in *MsYABBY5* RNAi (A) and overexpression plants (B) Leaves from the second node (2–3 cm) were harvested and used for qPCR analysis. Gene expression was normalized against the house keeping gene *ef1*. *P < 0.05; **P < 0.01. (C) MsNTT was localized to the chloroplast membrane in *Nicotiana benthamiana*.

To better understand MsYABBY5's biological functions and signalling pathways, it is essential to know its downstream target genes. There is not much known about the molecular mechanism of regulation by *YABBYs* and their direct target genes. As genes encoding enzymes in the biosynthetic pathways leading to monoterpene production were not significantly changed, we decided to investigate the expression of genes for transporters. Transporters play a key role in plant cellular metabolism (Fischer, 2011; Flügge *et al*., 2011). From our previous RNA‐Seq data, we had identified several transcripts in PGT which were involved in transport of carbon and ATP (Jin *et al*., 2014). One such transcript *MsNTT* similar to the plastidic ATP/ADP transporter‐like proteins showed differential expression in overexpression and RNAi plants. All the RNAi lines showed increased *MsNTT* transcript levels compared with WT plants or *35S::GFP* control plants and reduced expression was observed in the overexpression plants (Figure 7A,B). Plastidic ATP/ADP transporter facilitates the movement of ATP across the plastid inner membrane and can determine the rate of metabolic activity in plastids (Flügge *et al*., 2011; Neuhaus *et al*., 1997). MsNTT was seen localized to the plastid membrane (Figure 7C). Enhanced energy import into the plastids can be one of the reasons the knock‐down plants showed a higher metabolic activity.

**Figure 8:**
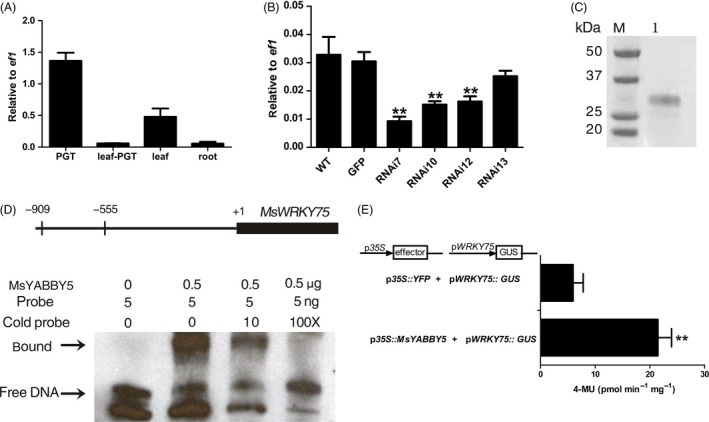
Transactivation of *MsWRKY75 by MsYABBY5*. (A) Expression pattern of *MsWRKY75* in different tissues of wild‐type spearmint. (B) Transcript levels of *MsWRKY75* in *MsYABBY5* RNAi plants. (C) Purification of recombinant His tagged MsYABBY5 expressed in *Escherichia coli*. (D) EMSA assay of MsYABBY5 binding ability to *MsWRKY75* promoter. Upper panel shows the fragment −909 to −555 of *MsWRKY75* promoter region, (E) transactivation of *MsWRKY75* by MsYABBY5 in *Nicotiana Benthamiana*. Results are presented as mean ± SD. **, *P* < 0.01.

In a recent study, ChIP‐Seq and RNA‐Seq methods were used to identify YABBY‐regulated genes during various stages of soya bean seedling development. The major candidate genes regulated by YABBY were found to be fatty acid desaturase, APETALA2 (AP2) and WRKY transcription factor (Shamimuzzaman and Vodkin, 2013). Recent research shows the emergence of WRKY TFs as key regulators of terpene production (Patra *et al*., 2013). From our RNA‐Seq data, we identified a WRKY transcript *MsWRKY75*, which was enriched in PGTs (Figure 8A). The level of this transcript showed reduction in *MsYABBY5* RNAi lines, but no significant increase was observed in overexpression lines (Figure 8B). To check whether MsYABBY5 can bind to the promoter regions of *MsNTT* and *MsWRKY75*, we performed EMSA using the purified recombinant His tagged MsYABBY5 protein (Figure 8C). About ~1 kb promoter regions of both the genes were cloned and they were divided into four overlapping fragments and screened by EMSA. No binding was observed with *MsNTT* promoter, but a protein–DNA complex with reduced mobility was observed when recombinant MsYABB5 was incubated with *MsWRKY75* probe (Figure 8D). Interestingly, only the fragment of −909 to −555 bp region of *MsWRKY75* promoter was found to bind with MsYABBY5. DNA binding specificity was further confirmed by competition experiments using 10–100‐fold excess unlabelled probe which led to the disappearance of DNA/protein complex. To further determine whether MsYABBY5 protein can regulate the *MsWRKY75* promoter in plants, transient expression assays in *N. benthamiana* were performed. Leaves were coinfilterated with reporter *MsWRKY75 promoter::GUS* and effector *35S::YFP* or *35S::MsYABBY5*. Promoter activity of *MsWRKY75::GUS* was significantly enhanced in 35S*::MsYABBY5* expressing leaves when compared to *35S::YFP* (Figure 8E). This suggests that MsYABBY5 activates *MsWRKY75* which probably represses terpene production in spearmint.

### Ectopic expression of *MsYABBY5* affects secondary metabolism in tobacco and sweet basil

**Figure 9:**
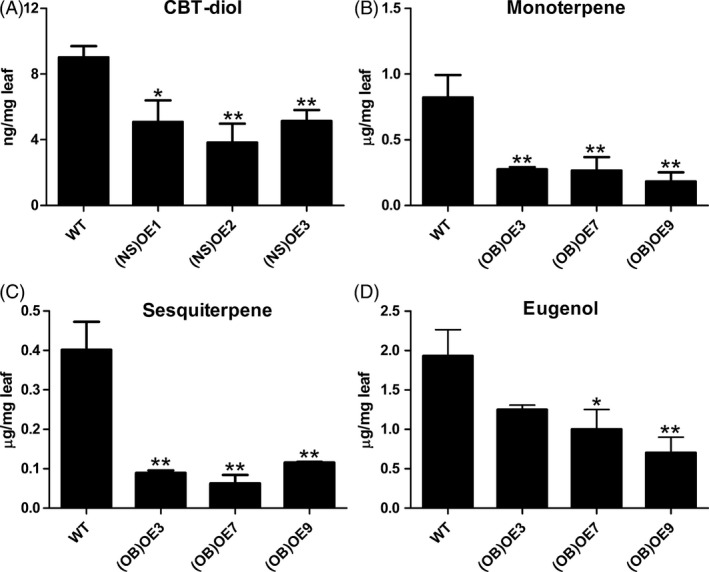
Ectopic expression of *MsYABBY5* decreased terpene production in *Nicotiana sylvestris* and sweet basil. (A) CBT‐diol production in wild‐type and transgenic *N. sylvestris* overexpressed with *MsYABBY5*. Volatiles production of total monoterpenes (B), sesquiterpenes (C) and eugenol (D) in wild‐type and transgenic basil overexpressed with *MsYABBY5*. Leaves from the second node (2–4 cm) were harvested and used for GC–MS analysis. Results of terpenes and phenylpropanoid production are presented as mean ± SD. *P < 0.05; **P < 0.01.

To understand the functions of *MsYABBY5* in other plants, the gene was ectopically expressed in tobacco and sweet basil. High transcript levels of *MsYABBY5* were detected in transgenic tobacco and sweet basil plants (Figure S4). *Nicotiana sylvestris* glandular trichomes mainly produce diterpenes which are generally derived from the same MEP pathway as the monoterpenes. Ectopic expression of *MsYABBY5* was found to reduce cembranoids (CBT‐diol) production in *N. sylvestris* by 29.5%–47.1% (Figure 9A). Sweet basil essential oil produced in PGT consists of both terpenes and phenylpropanoids. To explore whether *MsYABBY5* has an effect on secondary metabolites originating from different metabolic pathways in PGT, we ectopically expressed *MsYABBY5* in sweet basil. Three independent sweet basil transgenic lines confirmed by southern hybridization were selected for further characterization. The results of qRT‐PCR showed high expression levels of *MsYABBY5* in all the transgenic plants (data not shown). GC–MS analysis on T‐2 plants showed that the total production of both monoterpene (eucalyptol, β‐ocimene and linalool) and sesquiterpene (α‐bergamotene, γ‐muurolene and copaene) decreased (Figure 9B,C). Besides terpene production, phenylpropanoid production was also affected. Eugenol, which is the dominant compound, with a production of 1.93 ± 0.58 μg/mg fresh leaf, showed a significant reduction (*P *< 0.05) in transgenic plants (Figure 9D). This suggests that transcriptional regulators can govern fluxes in multiple metabolic pathways. Additionally, the sweet basil transgenic lines also showed curled leaf and delayed flowering (about 2–3 weeks delay) when compared to WT plants sown at the same time (Figure S5).

## Discussion

Glandular trichomes are found on the aerial surface of approximately 30% of vascular plants. As they can synthesize and store a large amount of secondary metabolites, they are aptly termed as ‘tiny chemical factories’ of plants. But very few studies have focussed on TFs that regulate glandular trichome‐specific metabolic pathways (Wang, 2014), which will greatly facilitate metabolic engineering efforts to increase yield or develop plant platforms to produce high value compounds. Studies in understanding the transcriptional control of secondary metabolite production show the expression of both activators and repressors is necessary to fine‐tune the flux, timing and the level of structural gene expression in a pathway (Albert *et al*., 2014; Cavallini *et al*., 2015; Patra *et al*., 2013). In this study, we isolated a *YABBY* gene that is preferentially expressed in spearmint PGT and appears to be a negative regulator of secondary metabolism. This is a novel function for the plant‐specific *YABBY* family of TFs. In Arabidopsis, *YABBY* gene family promotes several aspects of leaf, shoot and flower development (Eshed *et al*., 1999; Goldshmidt *et al*., 2008; Golz *et al*., 2004; Stahle *et al*., 2009), but how they mediate these effects at the molecular level largely remains unknown. Single mutants of *yabby5* in Arabidopsis showed no morphological defects, but significantly enhanced the phenotype of *yab1yab3* double mutant (Sarojam *et al*., 2010). In rice, the functions of *YABBY* genes are divergent from their Arabidopsis homologs. Rice *YAB1* is required for gibberellin‐mediated repression of *GA3ox2* gene which is involved in the synthesis of gibberellin (Dai *et al*., 2007).

Studies have revealed that YABBYs are bifunctional TFs acting as either repressors or activators (Bonaccorso *et al*., 2012; Stahle *et al*., 2009), but their direct downstream target genes are not well known. The increase in monoterpene production observed in *MsYABBY5* RNAi lines was not due to an increase in transcripts level of the structural genes involved in the pathway. Additionally, no significant changes were observed in transcript level of genes encoding enzymes in the precursor MEP pathway. The difference between metabolite and transcript levels can be attributed to either post‐transcriptional modification, protein stability or enhanced flux into the metabolic pathway (Xie *et al*., 2008). In peppermint, it was shown that most of the biosynthetic enzymes leading to monoterpene production including limonene synthase were regulated at the level of gene expression (McConkey *et al*., 2000). Many primary metabolic pathways like glycolysis, the TCA cycle, pentose phosphate pathway and shikimate pathway provides carbon, ATP and precursors compounds to diverse secondary metabolic pathways. Transcription factors can affect the synthesis of a particular secondary metabolite by regulating metabolic enzymes in these primary pathways (Aharoni and Galili, 2011). Both mint and basil PGT are nonphotosynthetic organs and need to import ATP and carbon to sustain their high metabolic activities, which too can be possibly controlled by TFs. The fact that *MsYABBY5* expression was able to affect metabolite production in tobacco and sweet basil plants suggests that this gene might be probably functioning upstream regulating flux into metabolic pathways. The MEP pathway and shikimate pathway leading to mono/diterpene and phenylpropanoid precursor production, respectively, are both localized in plastids making direct interactions between these pathways possible. *Production of anthocyanin pigment* (*PAP1*), a MYB transcription factor from Arabidopsis, is an activator of the phenylpropanoid pathway (Li *et al*., 2010). Recently it was shown that ectopic expression of *PAP1* in rose plants, led to an increase in floral volatile compounds originating from both phenylpropanoid and terpenoid pathways. Transcriptional activation of only few biosynthetic genes was observed, whereas the rest of the increase was attributed to enhanced flux in both pathways (Ben Zvi *et al*., 2012). Interactions between phenylpropanoid and terpenoid pathways have also been shown in tomato mutants (Enfissi *et al*., 2010), as well as in Ipomoea flowers (Majetic *et al*., 2010), but the mechanism still remains to be elucidated.

In a recent study, ChIP‐Seq and RNA‐Seq methods were used to identify YABBY‐regulated genes during various stages of soya bean seedling development. About 96 potential genes were found to be either up‐regulated or down‐regulated by YABBY. One of the major candidate genes regulated by YABBY was found to be WRKY transcription factor (Shamimuzzaman and Vodkin, 2013). WRKY TFs play important roles in regulation of plant stress response and secondary metabolism both as activators and repressors (Schluttenhofer and Yuan, 2015). Research has shown that WRKYs can activate structural genes involved in monoterpene (Spyropoulou *et al*., 2014), sesquiterpene (Ma *et al*., 2009) and diterpene (Qiu *et al*., 2008), as well as phenylpropanoid production (Wang *et al*., 2007). On the other hand, they can act as negative regulators too. In rice, *OsWRKY76* repressed terpene and phenylpropanoid synthesis but increased cold stress tolerance (Yokotani *et al*., 2013). In rubber, *HbWRKY1* negatively regulates a gene involved in natural rubber synthesis (Zhou *et al*., 2012).

In our study, we found that *MsYABBY5* negatively regulates the process of terpene biosynthesis and activates *MsWRKY75* in spearmint. MsWRKY75 may be a negative transcriptional regulator of genes involved in terpene synthesis. *MsYABBY5* expression overlaps with *MsWRKY75* in PGTs. Our RNA seq was performed on PGTs isolated from young leaves at a stage where terpene production is very active. The high expression of both of these TFs at this stage does indicate an important role for them. In *MsYABBY5* overexpression lines, no significant increase in *MsWRKY75* was observed; this can be due to a threshold of minimal expression required for *MsWRKY75* activation. *MsWRKY75* probably is one of the many downstream targets regulated by *MsYABBY5* to control secondary metabolism. Transcriptome analysis of *MsYABBY5* RNAi lines can provide us with more candidate genes that are potentially regulated by *MsYABBY5*. Our data reveal a regulatory network involving *YABBY5* and *WRKY* that controls terpene production in spearmint. As information regarding terpene pathway regulation is minimal, where *YABBY5* and *WRKY75* fall in the regulatory hierarchy to govern terpene biosynthesis and how they function with other TFs in the network remains to be explored. Considering the phenotypes observed in transgenic plants, it seems plausible that *MsYABBY5* controls an upstream event in metabolite production.

We found increased levels of transcript for the plastidic ATP/ADP transporter in transgenic lines making more secondary metabolites and decreased levels in transgenic lines producing less metabolite. In potato, overexpression and suppression of the plastidic ATP/ADP transporter led to an increase and decrease, respectively, in the amount of tuber starch produced (Tjaden *et al*., 1998). Further overexpression of both NTT and glucose‐6‐phosphate/phosphate translocator which supplies carbon skeletons to the plastids significantly increased total starch content in potato (Zhang *et al*., 2008). This suggested that import of both energy and carbons into amyloplasts is a rate‐limiting step for starch formation. Most of the plastid proteins are encoded in the nucleus and transported to plastids. *MsYABBY5* does not seem to directly regulate the expression of *MsNTT* to control secondary metabolite production, but the effect can be indirect due to changes in secondary metabolism brought by *MsYABBY5*.

Immunostaining and fluorescence assays showed MsYABBY5 localized to both nucleus and cytoplasm. Cell fractionation and protein gel blot assays can be further performed to test the association of MsYABBY5 with intracellular membranes. There are several reports of TFs especially those involved in stress response to rapidly translocate from the cytoplasm to the nucleus in response to external signals. Some examples from the mammalian systems are STAT (signal transducer and activator of transcription), NF‐κB (nuclear factor of immunoglobulin kappa B cells), NFAT (nuclear factor of activated T cells) and steroid receptors proteins (Beals *et al*., 1997; McBride *et al*., 2002). In the case of plants, ER membrane‐associated bZIP and NAC089 TFs which are responsible for mediating ER‐related plant immunity and abiotic stress responses, respectively, were detected in the cytoplasm (Che *et al*., 2010; Moreno *et al*., 2012; Yang *et al*., 2014). Abscisic acid (ABA) is a vital plant hormone that plays important roles in stress response. In Arabidopsis, a WRKY protein, AtWRKY40 functions as negative regulator of ABA signalling by directly repressing expression of many ABA‐responsive genes. WRKY40 was shown to localize to both nucleus and cytosol in wild‐type plant cells that has ABA at physiological concentrations and ABA was essential for its cytosolic distribution (Shang *et al*., 2010). Secondary metabolites produced by plants are also related to abiotic and biotic stress responses of plants. Further studies are required to assess the change in expression levels and localization patterns of MsYABBY5 and WRKY75 in response to stress conditions and its significance.

## Experimental procedures

### Plant material and transformation

Commercial spearmint variety (*M. spicata*) and sweet basil (*O. basilicum*) were tested for their secondary metabolites by GC–MS and grown in greenhouse under natural light conditions. Spearmint plants were propagated using stem cuttings, whereas basil plants were propagated from seeds. *Agrobacterium‐*mediated transformation of spearmint was performed according to the previously published protocol (Niu *et al*., 1998, 2000). *Agrobacterium*‐mediated transformation of sweet basil was performed by the following procedure. *O. basilicum* seeds were sterilized by washing in 40% Clorox for 3 min followed by several rinses with sterile water. The sterile seeds were imbibed overnight and kept at 4 °C. The following day the seeds were dissected under a dissection microscope to harvest the mature embryos. The dissected embryos were precultured in dark for 1 day in cocultivation medium (CC). *Agrobacterium* EHA105 strain was used for transformation. Enhanced green‐fluorescent protein gene along with kanamycin was used as a selection marker. The precultured embryos were immersed in agrobacterium culture and sonicated for 15 s, four times. After sonication, the embryos were immersed in fresh *Agrobacterium* solution and vacuum‐infiltrated for 3 min. After infection for 30 min, the embryos were placed in CC medium [MS salts + myo‐inositol 100 mg/L sucrose (30 g/L) + BA (0.4 mg/L) + IBA (0.4 mg/L) + cefotaxime (150 mg/l)] for 3 days. After 3 days, the embryos were washed multiple times with sterile‐distilled water containing cefotaxime (150 mg/L). The washed embryos were kept in CC media for 3–4 weeks in dark for shoot induction. After 3–4 weeks, GFP‐positive shoots were selected and transferred to light. The well‐grown shoots were transferred to elongation media [MS salts + sucrose (30 g/L) + BA (3 mg/L) + IAA (0.5 mg/L) + cefotaxime (150 mg/L)] and kept for 2–3 weeks. The shoots were hardened on basal media and allowed for root formation. Plantlets with well‐developed roots were transferred to soil and grown under glasshouse conditions before further analysis. Tobacco transformation was done as previously described by Gallois and Marinho (1995).

### RNA extraction and quantitative RT‐PCR

Total RNA was extracted from different tissues (PGT, leaf‐PGT, leaf and root) of spearmint using an RNeasy^®^ Plus Mini kit from Qiagen. Reverse transcription reaction and quantitative RT‐PCR (qRT‐PCR) were carried out as described in Jin *et al*. (2014). Expression levels of target genes were represented as mean ± SD. Approximately 1 μg RNA was employed to synthesize first strand cDNA.

### *In situ* hybridization

*In situ* hybridization assay was performed according to the method described by Javelle *et al*. (2011) with some minor modifications. Briefly, samples were fixed in 4% paraformaldehyde fixative and subjected to vacuum for 30 min on ice. After that, the vials were kept overnight at 4 °C. Next day, samples were dehydrated with ethanol series and embedded in Paraplast (McCormick Scientific, St Louis, MO) until use. The blocks were sectioned at 10 μm and mounted on Probe‐on Plus slides (Fisher Scientific, singapore). For probe synthesis, the *MsYABBY5* cDNA was inserted into a pGEM^®^‐T vector (Promega, Wisconsin). Sense and antisense probes were synthesized by T7 and SP6 RNA polymerase (Roche, Basel, Switzerland), respectively.

### Cloning and vector construction

#### Promoter cloning *of MsYABBY5*

Genomic DNA was isolated from young leaves of spearmint using CTAB method. The flanking sequence of *MsYABBY5* gene was amplified using a GenomeWalker^™^ Universal kit. The −1116 bp flanking region of the gene was ligated with pGEM^®^‐T vector. The resulting product was transformed into *Escherichia coli* XL1‐Blue and sequenced. The promoter was amplified with Phusion^®^ High‐Fidelity DNA Polymerase (NEB) and subcloned into a gateway donor vector pENTR^™^/D‐TOPO^®^ (Invitrogen, California). Then, the recombinant plasmid was introduced into destination vectors pBGWFS7 by LR recombination. The destination plasmid was further transformed into *A. tumefaciens* EHA105 by heat shock and used to generate transgenic tobacco lines. Sequences of all primers used in this study are listed in Table S1.

#### Full‐length cloning of all four *YABBYs*

Full length of all four YABBYs cDNAs was obtained by performing 3′ and 5′ RACE using the SMARTer^™^ RACE cDNA amplification kit from Clontech. For sequencing full‐length ORFs, the purified fragments were ligated with pGEM^®^‐T vector. The resulting product was transformed into *E. coli* XL1‐Blue.

### Overexpression and RNAi vector construction

To overexpress or silence *MsYABBY5*, sequences were amplified with Phusion^®^ High‐Fidelity DNA Polymerase (NEB). The purified fragments were inserted into a gateway donor vector pENTR^™^/D‐TOPO^®^ (Invitrogen). Then, the recombinant plasmids were introduced into destination vectors pK7WG2D for overexpression in spearmint and sweet basil by LR recombination. For *MsYABBY5* RNAi, four primers with restriction enzymes located at flanking region were used to amplify the fragment showing low similarity to other *YABBY* genes. The purified PCR product was cloned into the donor vector and subsequently introduced into pK7WG2D by LR recombination. The *MsYABBY5* gene was driven by *35S* promoter in both overexpression and RNAi plants. All destination plasmids harbouring the target genes were transformed into *A. tumefaciens* EHA105 by heat shock and used for spearmint and basil transformation.

### Subcellular localization

*YABBY* and *MsNTT* ORFs were amplified and inserted into the pENTR^™^/D‐TOPO^®^. The donor vectors harbouring ORFs were introduced into pBADC/YFP vector by LR recombination. For testing expression pattern of *MsYABBY5* promoter, the 5′UTR sequence was amplified and inserted into pENTR^™^/D‐TOPO^®^. Subsequently, the plasmid was transformed into pBGWFS7 by LR recombination. All destination plasmids harbouring target genes were transformed into *A. tumefaciens* EHA105 by heat shock. The recombinant *A. tumefaciens* EHA strains were used for plant transformation. Subcellular localization pattern of YABBY proteins was performed as described in Jin *et al*. (2014). Briefly, the recombinant *A. tumefaciens* EHA strains were grown in LB medium overnight at 28 °C. After centrifugation at 4000 × ***g***, 4 °C for 15 min, cell pellets were collected and resuspended in MMA solution (10 mm MES, 10 mm MgCl_2_, 100 μm acetosyringone) to OD_600_ = 1. The solution was then injected into *N. benthamiana* leaves. After that, plants were kept at 28 °C for 2 days. Leaf samples were collected and viewed with an upright confocal microscope (Zeiss, Jena, Germany).

### Southern blotting

A total of 15 μg genomic DNA was digested overnight with *Eco*RI at 37 °C. Next day, digestion product was electrophoresed on a 1.2% (w/v) agarose gel at 50 V for 4 h. After that, the gel was transferred to a nylon membrane and hybridized by the CaMV *35S* promoter probe using a DIG DNA labelling and detection kit (Roche). DNA probe against *35S* promoter was generated using PCR DIG probe synthesis kit from Roche (Hart and Basu, 2009).

### Immunogold labelling

A 14‐AA peptide of MsYABBY5 showing low similarity to other MsYABBYs was used as antigen for antibody synthesis (GenScript, Piscataway, NJ). Specificity test of the antibody was performed by Western blotting). Leaf samples from spearmint were fixed for 3 h in 4% paraformaldehyde/0.5% glutaraldehyde in 0.1 M phosphate buffer (pH 7.2) and rinsed in 0.1 M phosphate buffer (pH 7.2) for three times followed by dehydration in ethanol. After that, the samples were infiltrated with and embedded in LR White. Ultrathin sections around 90 nm were prepared with Leica Ultracut UCT microtome equipped with diamond knives and collected on uncoated, 300‐mesh nickel grids. The procedure of labelling and washing was performed according to the protocol described by Skepper and Powell (2008) with some minor modifications. Briefly, the sections were incubated for 4 h on drops of antimint YAB5 antibody (produced in rabbit) with 1 : 100 dilutions in PBSG buffer [1% (w/v) gelatin in PBS buffer]. After that, sections were rinsed on drops of TBST (50 mm Tris, 150 mm NaCl, 0.05% Tween 20) for ten times, 2 min for each time. Then, sections were incubated for 1 h on drops of goat anti‐rabbit antibody conjugated with 10 nm gold particles with 1 : 100 dilutions in PBSG buffer [1% (w/v) gelatin in PBS buffer]. The sections were further rinsed in TBST buffer for 10 times, 2 min each time, and in ddH_2_O for 30 s. Subsequently, samples were counterstained by applying the grid on drops of uranyl acetate and lead citrate. Finally, sections were extensively rinsed in ddH_2_O and viewed at 120 kV with a transmission electron microscope (JEOL JEM‐1230, Japan).

### Electrophoretic mobility shift assay (EMSA)

MsYABBY5 was expressed in *E. coli* BL21 (DE3) and induced by 1 mm isopropyl‐β‐thiogalactopyranoside (IPTG) for 6 h. Then, the recombinant protein was purified using 6× His tagged Ni‐NTA agarose (Qiagen, Hilden, Germany) and used for EMSA. The 5′ ends of probes used for EMSA were labelled with biotin (Table S1). The assay was performed using a LightShift Chemiluminescent EMSA kit (Thermo, Waltham, MA) according to the manufacturer's instructions.

### Transactivation activity assay

The ~1 kb promoter region of *MsWRKY75* was amplified and inserted into pENTR^™^/D‐TOPO^®^. The resulting plasmid was transformed into pBGWFS7 by LR recombination and further introduced into *A. tumefaciens* EHA. Leaves of *N. *b*enthamiana* were agroinfiltrated with effector and reporter at a ratio of 1 : 1. Two days after infiltration, leaves were harvested to isolate crude protein. GUS quantitative assay was performed as described by Li *et al*. (2014). Each assay was performed in triplicate.

### Gas chromatography–mass spectrometry analysis

In case of mint, each transgenic plant was propagated clonally before GC–MS studies were conducted on them. For basil and tobacco plants, the analysis was performed on T‐1 plants. Terpene and phenylpropanoid production in leaves of spearmint, sweet basil and tobacco were determined using a GC–MS method as described in Jin *et al*. (2014). Camphor was used as an internal standard.

### Statistical analysis

Data are indicated as ‘mean ± SD’ of three biological replicates each performed in triplicates. Statistical significance between transgenic plants and WT was analysed using a two‐tailed Student's *t*‐test and indicated by asterisks. *P < 0.05; **P < 0.01.

## Competing interests

The authors declare that they have no competing interests.

## Supporting information


**Figure S1** MsYABBY5 protein was observed in both nucleus (N) and cytoplasm (C) in peltate glandular trichome (PGT) of spearmint.
**Figure S2** Southern blotting analysis of transgenic plants.
**Figure S3** Transcript levels of other *MsYABBYs* in RNAi plants.
**Figure S4** Transcript level of *MsYABBY5* in *MsYABBY5* overexpression sweet basil (A) and *Nicotiana sylvestris* (B).
**Figure S5** Ectopic expression of *MsYABBY5* caused leaf curling and flowering time delay in sweet basil.
**Table S1** Sequences of the primers used in this study.
