# Supplementary material for: Metabolic engineering of terpene biosynthesis in plants using a trichome‐specific transcription factor MsYABBY5 from spearmint (Mentha spicata)
Source: Plant Biotechnol J. 2016 Feb 4;14(7):1619–32. doi: 10.1111/pbi.12525 (PMC5067620; doi:10.1111/pbi.12525)
Supplement: Supplementary file 1 — Figure S1 MsYABBY5 protein was observed in both nucleus (N) and cytoplasm (C) in peltate glandular trichome (PGT) of spearmint. Figure S2 Southern blotting analysis of transgenic plants. Figure S3 Transcript levels of other MsYABBYs in RNAi plants. Figure S4 Transcript level of MsYABBY5 in MsYABBY5 overexpression sweet basil (A) and Nicotiana sylvestris (B). Figure S5 Ectopic expression of MsYABBY5 caused leaf curling and flowering time delay in sweet basil. Table S1 Sequences of the primers used in this study. [file PBI-14-1619-s001.pdf]

Support information

Table S1. Sequences of the primers used in this study[1]

Name	Sequence (5' to 3')	purpose
YAB5-5GSP1	CACACCTAACTGTCACTACAT	RACE of Msyabby5
YAB5-5GSP2	TCCGATGGAGCGTAGCTAAGAC	RACE of Msyabby5
YAB5-3GSP1	TCATGCTGGAGAGCAAGAACCAAG	RACE of Msyabby5
Qyab5-F1	GTGACAGTTAGGTGTGGGCA	qPCR of Msyabby5
Qyab5-R2	CCATTTGGAAGAGGAGCCGA	qPCR of Msyabby5
YAB-GW-5GSP1	CAAGGAATGTAACACAGCTGCTCAGCC	genome walking of Msyabby5
YAB-GW-5GSP2	GCCCACACCTAACTGTCACTACATCAA	genome walking of Msyabby5
yab5'UTR(-1166)	CACCACTTAACGGTTCTTTCAGGACC	cloning of Msyabby5 promoter
yab5'UTR(-10)	TCAAGGAATTCGAAAAAGAGGAAG	cloning of Msyabby5 promoter
yabOE-F	CACCATGGATATGGCTGAGCAGC	over expression of Msyabby5 in plant
yabOE-R	TTTGTTCAGAACGGCTGCCCTT	over expression of Msyabby5 in plant
yab5-SphI	CGCATGCACGCCTCCTCCTTCCAAGAT	silencing of Msyabby5
yab5-BamHI	CGGATCCAACGGCTGCCCTTCTCATTT	silencing of Msyabby5
yab5-XbaI	CTCTAGAACGCCTCCTCCTTCCAAGAT	silencing of Msyabby5
yab5-XhoI	GCTCGAGAACGGCTGCCCTTCTCATTT	silencing of Msyabby5
YAB5-BamHI2	CGGATCCCATGGATATGGCTGAGCAGC	Over expression of Msyabby5 in *E.coli*
YAB5-XhoI2	ACTCGAGTTTGTTCAGAACGGCTGCCCTT	Over expression of Msyabby5 in *E.coli*
MsYABBY6-5GSP	CATTAAGATTAGGTTGGGTATTTGACTCC	RACE of Msyabby6
MsYABBY6-3GSP	GGAGTCAAATACCCAACCTAATCTTAATG	RACE of Msyabby6
MsYABBY2-5GSP	CCGCTAAAATGGTGTTGCAGTAGTTG	RACE of Msyabby2
MsYABBY2-3GSP	GATAGCAACAAACAAGCAAAACTGGAT	RACE of Msyabby2
MsYABBY4-5GSP	AATGAATACGAGGAAGATGTGCCCAAT	RACE of Msyabby4
MsYABBY4-3GSP	AGGAGATTCAGAGGATAAAGGCTAGCA	RACE of Msyabby4
MsYABBY6_c1F	ACAGCACACTCGAAGACATA	qPCR of Msyabby6
MsYABBY6_c1R	AACCTAATCTTAATGAGGGG	qPCR of Msyabby6
MsYABBY2_c0F	TTGCTTGTTTGTTGCTATCC	qPCR of Msyabby2
MsYABBY2_c0R	GCTTGACACTGAACAACCCA	qPCR of Msyabby2
MsYABBY4_c1F	CACATCTTCCTCGTATTCATT	qPCR of Msyabby4
MsYABBY4_c1R	CAAAATAAGTGGCATCAAATC	qPCR of Msyabby4
MsYABBY6-OE-F	CACCATGGATGCCTCTGAGCAAAT	Subcellular localization of MsYABBY6
MsYABBY6-OE-R	TTTGTTTCGTACAGTCGCCCTTC	Subcellular localization of MsYABBY6
MsYABBY2-OE-F	CACCATGTCAATGGAATTGACGGCAG	Subcellular localization of MsYABBY2
MsYABBY2-OE-R	AAAACCAAGAGATTTTTGAGCGG	Subcellular localization of MsYABBY2
MsYABBY4-OE-F	CACCATGTCAGTTGATATGACTTTGG	Subcellular localization of MsYABBY4
MsYABBY4-OE-R	GCTTGTTTTGTTGAGTCCAAA	Subcellular localization of MsYABBY6

MsYABBY6-BamHI	CGGATCCCATGGATGCCTCTGAGCAAAT	over expression of Msyabby6 in *E.coli*
MsYABBY6-XhoI	ACTCGAGTTTGTTTCGTACAGTCGCCCTTC	over expression of Msyabby6 in *E.coli*
MsYABBY2-BamHI	CGGATCCCATGTCAATGGAATTGACGGCAG	over expression of Msyabby2 in *E.coli*
MsYABBY2-XhoI	ACTCGAGAAAACCAAGAGATTTTTGAGCGG	over expression of Msyabby2 in *E.coli*
MsYABBY4-BamHI	CGGATCCCATGTCAGTTGATATGACTTTGG	over expression of Msyabby4 in *E.coli*
MsYABBY4-NotI	AGCGGCCGCGCTTGTTTTGTTGAGTCCAAA	over expression of Msyabby4 in *E.coli*
35S(591)-F	CTCAGAAGACCAAAGGGCTATT	probe synthesis for Southern blotting
35S(-34)-R	TGTTTGTTTTGTTGTGGTATTG	probe synthesis for Southern blotting
qWRKY75-F	AGTCTCGCACAAAGCTCTCATCT	qPCR of WRKY75
qWRKY75-R	TGGGATGAAGCCCACGTGTC	qPCR of WRKY75
WRKY75-GW-5GSP1	TTGATGAAGAAGAAGGTTGGGAATAGT	genome walking of WRKY75
WRKY75GW-5GSP2	ATCGTGTGGTTGGGAGTTCATCATCA	genome walking of WRKY75
WRKY75(-909-)F	CACCTTGTCACGTGTTCATAATATTTCT	cloning of WRKY75 promoter
WRKY75(-1)-R	GAGAGAGAAATTAAGAGAAGAAAA	cloning of WRKY75 promoter
W75P-R1	GAAGAAAAAGAATGTCCAAGGA	probe synthesis for EMSA
qNTT1-F	CTCTTTGGGCTAGGTGCGAA	qPCR of MsNTT
qNTT1-R	TTCAAGGAGATGGCCCAACC	qPCR of MsNTT
NTT1-OE-F	CACCATGCAAGGTGTTCTTCAGTCA	subcellular localization of MsNTT
NTT1-OE-R	CACACTTCGGGGAGACGAGGG	subcellular localization of MsNTT

1 Restriction enzyme sites in primers were underlined. CACC overhangs for cloning to donor vector were shaded.

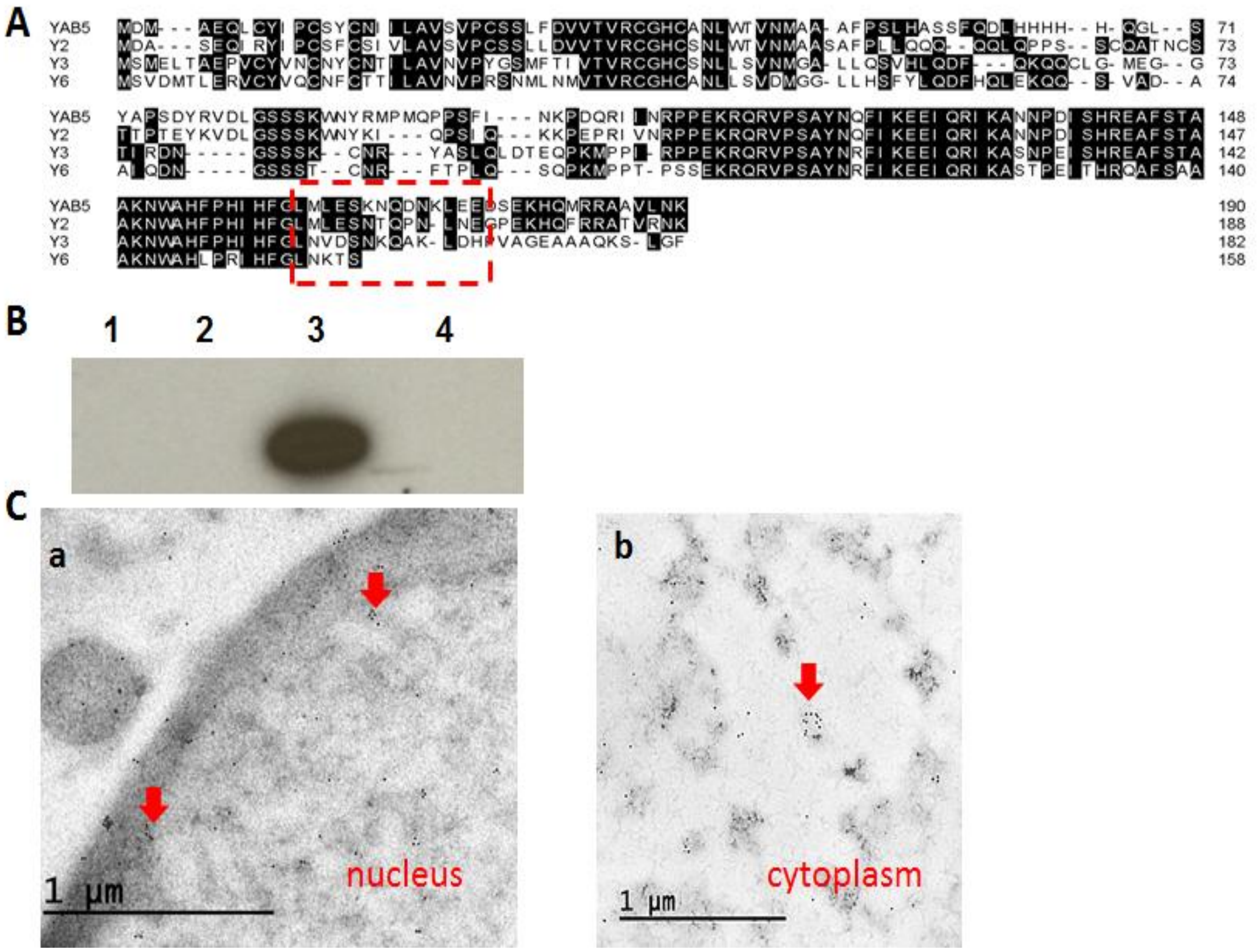

Figure S1. MsYABBY5 protein was observed in both nucleus (N) and cytoplasm (C) in peltate glandular trichome (PGT) of spearmint

A. Peptide with low similarity to other YABBY proteins was used for antibody synthesis. B. Specificity test of MsYABBY5 antibody. 1, MsYABBY2; 2, MsYABBY4; 3, MsYABBY5; 4, MsYABBY6. C. Immunogold labelling analysis showed MsYABBY5 protein was found in nucleus (a) and cytoplasm (b) of PGT.

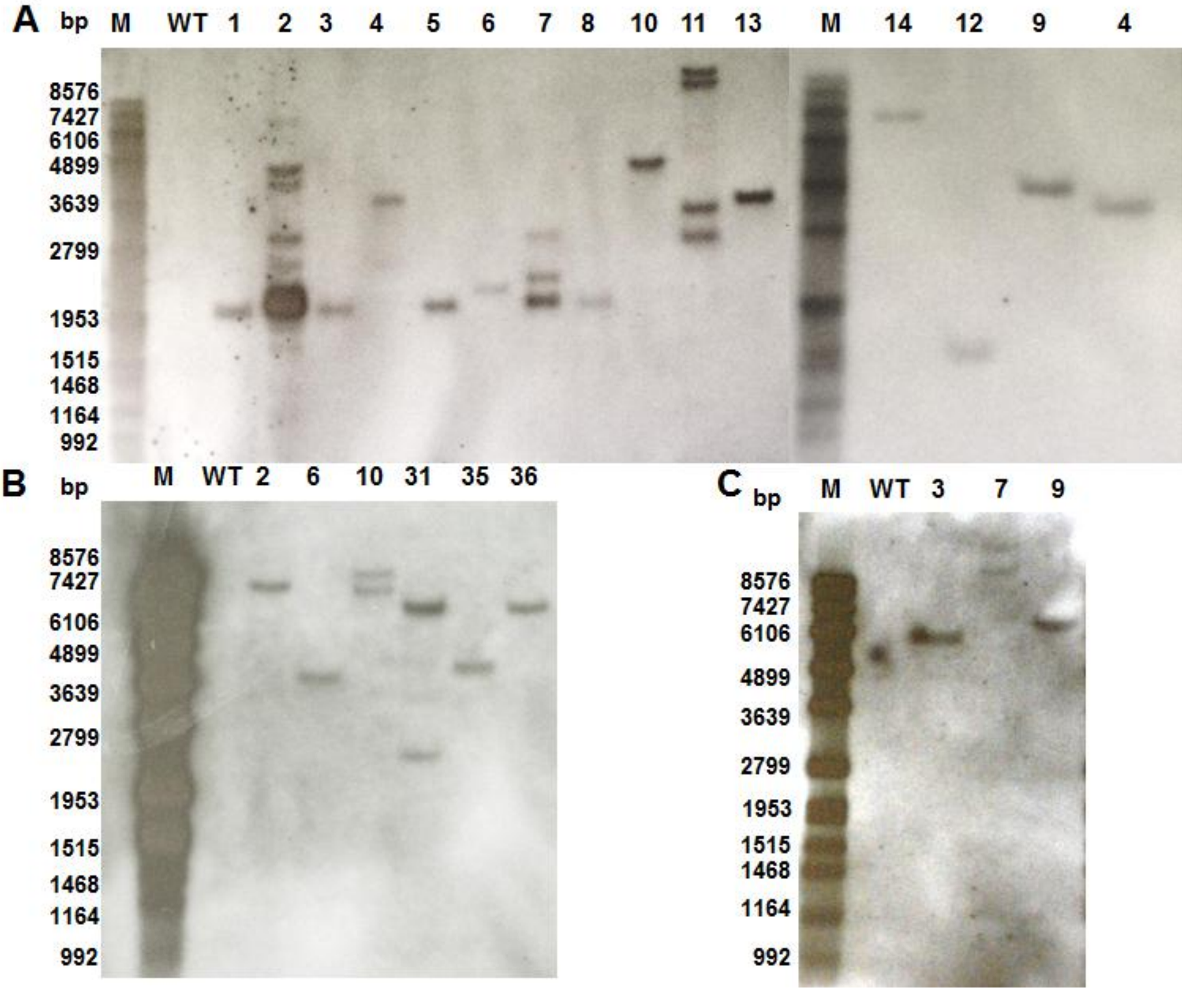

Figure S2. Southern blotting analysis of transgenic plants

A, *MsYABBY5* RNAi lines. B, over expression of Ms*YABBY5* in spearmint. C, over expression of *MsYABBY5* in sweet basil.

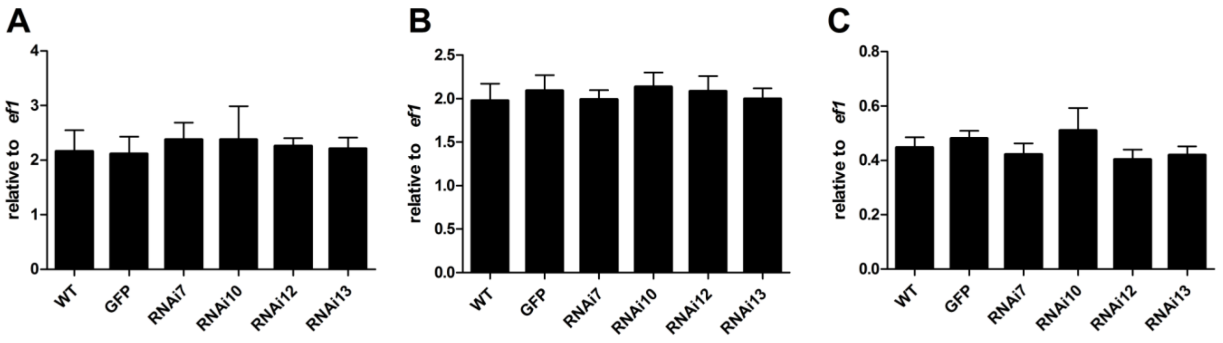

Figure S3. Transcript levels of other *MsYABBYs* in RNAi plants

A. *MsYABBY2*. B. *MsYABBY4.* C. *MsYABBY6.* Leaves from the second node (2-3 cm) were harvested and used for qPCR analysis. Gene expression was normalized against the house keeping gene *ef1*. *, *P*<0.05; **, *P*<0.01.

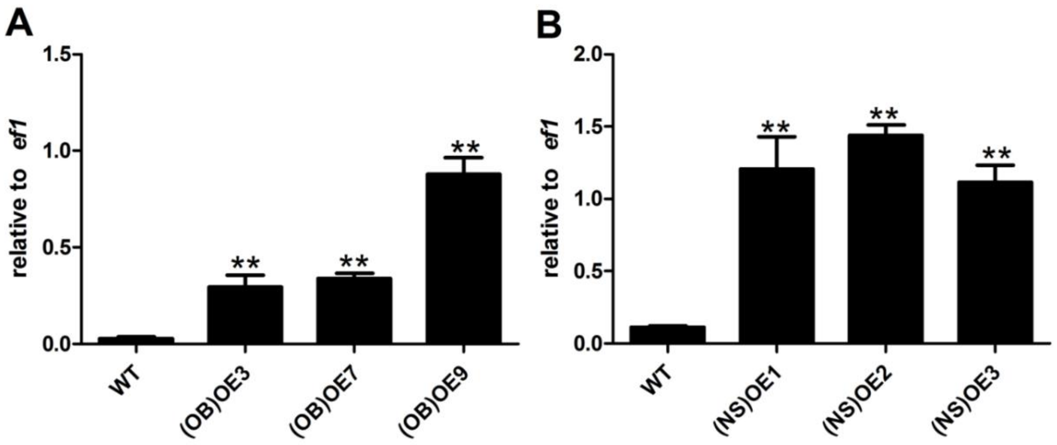

Figure S4. Transcript level of *MsYABBY5* in *MsYABBY5* overepression sweet basil (A) and *N. sylvestris* (B)

Leaves from the second node (2-4 cm) were harvested and used for qPCR analysis. Gene expression was normalized against the house keeping gene *ef1*. *, *P*<0.05; **, *P*<0.01.

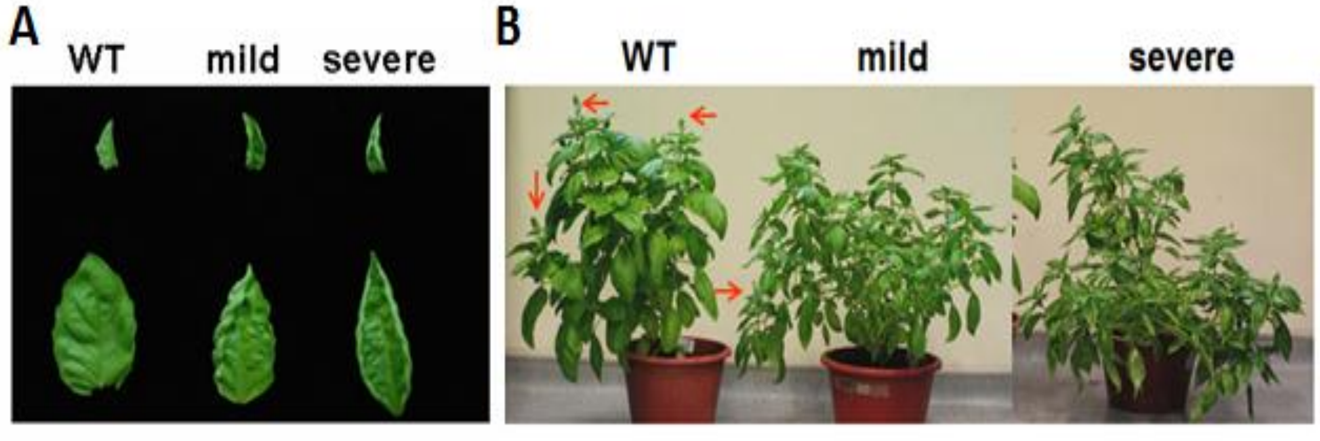

Figure S5. Ectopic expression of *MsYABBY5* caused leaf curling and flowering time delay in sweet basil

A. Ectopic expression of *MsYABBY5* caused curling of leaves in sweet basil. B. Delay of flowering time and curled leaves observed in *MsYABBY5* overexpression plants. Arrows indicate the inflorescence.
